# Supplementary material for: Exogenous Methyl Jasmonate (MeJA) Improves ‘Ruixue’ Apple Fruit Quality by Regulating Cell Wall Metabolism
Source: Foods. 2024 May 21;13(11):1594. doi: 10.3390/foods13111594 (PMC11171686; doi:10.3390/foods13111594)
Supplement: Supplementary file 1 [file foods-13-01594-s001.zip › foods-2977015-supplementary.pdf]

Supplementary Materials:

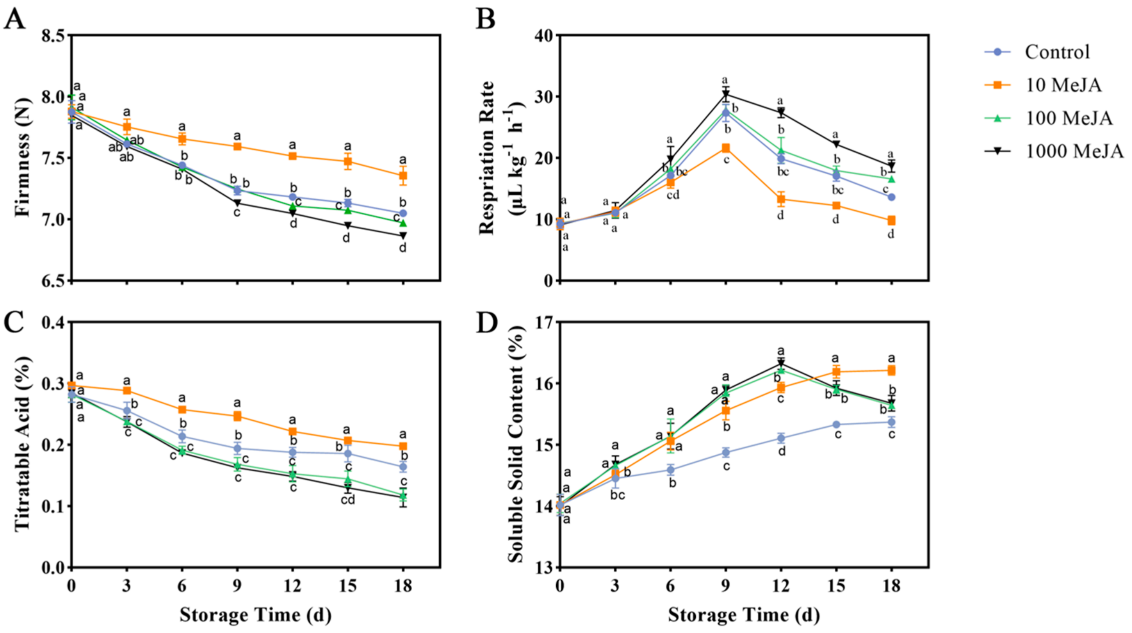

Figure. S1 Effects of MeJA treatment at different concentrations on quality traits of 'Ruixue' apple fruits. (A) Firmness, (B) Respiratory rate, (C) TA and (D) SSC.

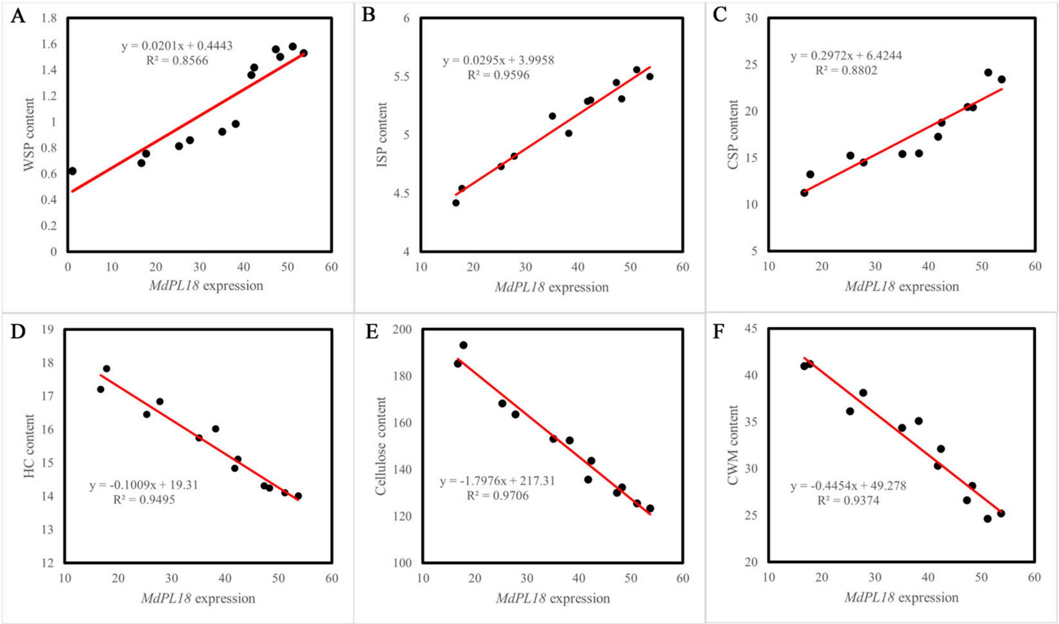

Figure. S2 Correlation analysis between content of WSP (A), ISP (B), CSP (C), HC (D), Cellulose (E), CWM (F) and expression levels of *MdPL18*.

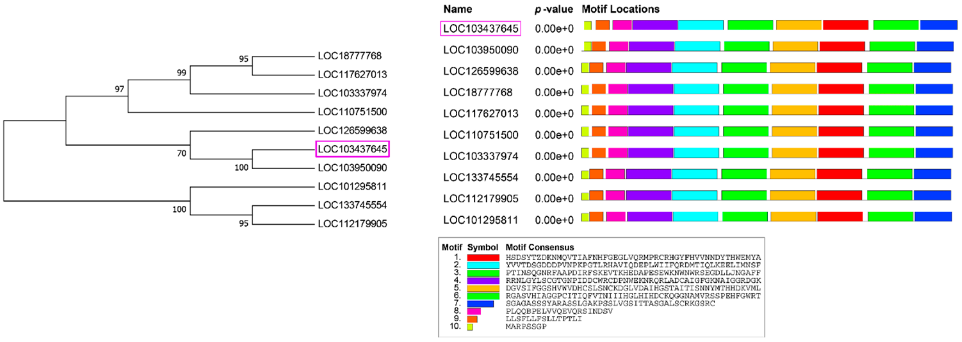

Figure. S3 Analysis of *MdPL18* family phylogeny and protein domains. (A) Maximum-likelihood phylogenetic tree reconstructed with MEGA 7.0, using 1000 bootstrap replicates. (B)Protein motifs. The motifs were detected using the MEME.

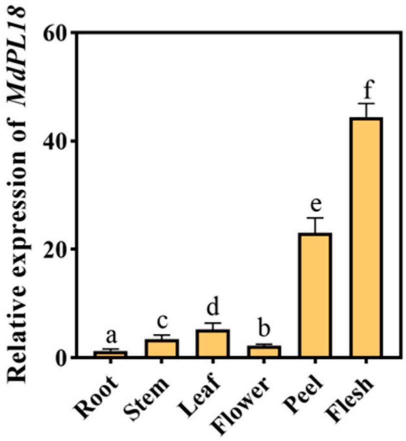

Figure. S4 The relative expression levels of the *MdPL18* gene in different organs. (*, $P < 0.05$; **, $P < 0.01$). Error bars show ± SE from three biological replicates.

Table. S1 Primer sequences used for qRT-PCR.

Primer name	Forward primer	Reverse primer
MdACS1	AGCCTCTCTAAGGATCTTGG	TGGTTCTCGGCTATGTAGTTCTT
MdACS2	ACGGGATTATTCAGATGGGTC	TGAGCACTAAGTGGTTGGGAT
MdACO1	GACTTGGACTGGGAAAGCAC	GGAGGGTAGTTGCTGACCTT
MdACO2	ACTCATTCATCAGCCCAGCAC	AAACCCAAACTTTCTTCTCCC
MdPL1	CTTACGGGGGAGGAGGGTG	TGGGCAATGTGCTGGAAGA
MdPL18	CATCCTGTAAACCCTAAACCCG	CTACCACCAAAAATGGAGACCC
Mdgal1	GGGAGTTCTTCTGTTGAATGGG	TTCCGGCATAAGAACAATCG
Mdgal18	CGAAATACCCTGTTAGAGTGAGC	CGATGAATGACTGCCAAGGAA
MdPG1	CGACTTCGCCACCACCGT	CCGAGCCCAAAGCCATAAA
